# Numaswitch: an efficient high-titer expression platform to produce peptides and small proteins

**DOI:** 10.1186/s13568-021-01204-w

**Published:** 2021-03-25

**Authors:** Bach-Ngan Nguyen, Florian Tieves, Thomas Rohr, Hilke Wobst, Felix S. Schöpf, Jóse D. Montoya Solano, Julia Schneider, Janpeter Stock, Andreas Uhde, Thomas Kalthoff, Karl Erich Jaeger, Lutz Schmitt, Christian Schwarz

**Affiliations:** 1 NUMAFERM GmbH, Merowingerplatz 1a, 40225 Düsseldorf, Germany; 2 Institute of Biochemistry, Heinrich-Heine University, 40225 Düsseldorf, Germany; 3 Institute of Molecular Enzyme Technology, Heinrich Heine University, Düsseldorf, Germany; 4 Institute of Bio- and Geosciences IGB-1: Biotechnology, Research Center Jülich, 52426 Jülich, Germany; 5 Department of Applied Synthetic Chemistry, TU Wien, 2060 Vienna, Austria

**Keywords:** Numaswitch, Switchtag, Recombinant peptides, High-titer peptide expression platform, Teriparatide, PTH(1-34), PTH(1-84), Liraglutide, Semaglutide, Active pharmaceutical ingredients

## Abstract

The production of peptides as active pharmaceutical ingredients (APIs) by recombinant technologies is of emerging interest. A reliable production platform, however, is still missing due the inherent characteristics of peptides such as proteolytic sensitivity, aggregation and cytotoxicity. We have developed a new technology named Numaswitch solving present limitations. Numaswitch was successfully employed for the production of diverse peptides and small proteins varying in length, physicochemical and functional characteristics, including Teriparatide, Linaclotide, human β-amyloid and Serum amyloid A3. Additionally, the potential of Numaswitch for a cost-efficient commercial production is demonstrated yielding > 2 g Teriparatide per liter fermentation broth in a quality meeting API standard.

**Supplementary Information:**

The online version contains supplementary material available at 10.1186/s13568-021-01204-w.

Peptides represent a class of active pharmaceutical ingredients (API) with rapidly increasing importance. About 5% of all APIs are peptides with a market volume of 25 billion US$ in 2018 and forecasted annually growth rates (CAGR) of 7.9% in 2019–2027 (Henninot et al. 2018; Muttenthaler et al. 2021; TMR 2020). Further applications are under development including (personalized) peptide vaccines, pesticides, cosmeceuticals, nutraceuticals, coatings, biosensors or antibiotic substitutions (Baratta 2019; Girija 2018; Karimzadeh et al. 2018; Lau and Dunn 2018; Mahlapuu et al. 2016; Pai et al. 2017; Schwinges et al. 2019; Townsend et al. 2017). Chemical synthesis and native chemical ligation are the dominant production strategies being reliable, quick to set-up and allowing the incorporation of non-natural amino acids (Dawson et al. 1994; Merrifield 1963). However, cost-of-goods, limited scalability, challenges with peptides > 20 aa and usage of harmful chemicals are major obstacles limiting a broader commercial application of peptides (Isidro-Llobet et al. 2019; Loibl et al. 2016).

A potent alternative are recombinant production approaches. However, associated challenges prohibit their usage, namely peptide proteolysis, degradation, aggregation and cytotoxicity towards the production host (Wegmuller and Schmid 2014). Various expression strategies were developed to circumvent such limitations based on the fusion with protein tags, for example inclusion body (IB) tags, solubility (S) tags or transport signals (TS) (Wegmuller and Schmid 2014). Being applied successfully for various peptides, the establishment of production processes is often time consuming and costly due to intrinsic disadvantages of each strategy. Being expressed as proteolysis resistant aggregates, typically in *E.coli* and at high titers, IB peptide fusions are challenging to be refolded hindering downstreaming steps and the production of functional products at high yields. Titers of cytoplasmically expressed S peptides are limited by the cell volume, targets might be proteolytically damaged, harm the producer cells and extensive downstreaming efforts are needed to reach high purity levels. The transport into the cell surrounding by Gram-positive bacteria or yeast is an alternative that is broadly applied for the industrial production of proteins. However, significant efforts are needed, i.e. for cloning, analysis of different signal sequences, definition of a suitable host and depletion of host proteases. Robust and efficient transport systems for the industrial standard host *E. coli* are not available. Recently, the first efficient secretion platform for *E. coli* was described (Khosa et al. 2018). However, reliability and applicability for peptides still need to be demonstrated.

In this study, we analyzed the protein hemolysin A (HlyA), the allocrite of the dedicated HlyA type 1 secretion system (T1SS) present in Gram-negative bacterium *E. coli,* to develop the Numaswitch approach. HlyA consists of three functional domains (Fig. 1); a hydrophobic N-terminal domain (hND), a Repeat-in-toxins (RTX) domain characterized by the presence of GG repeats (nonapeptide stretches of the consensus sequence GGXGXDXUX (where X can be any amino acid and U is a large hydrophobic amino acid) and a C-terminal secretion signal (SS, ~ 60 aa). The secretion signal is recognized by the T1SS inducing the secretion across the Gram-negative cell wall by the T1SS. After being secreted, Ca^2+^ ions bind to the GGs of the RTX domain inducing folding of HlyA into a soluble, stable and functional protein (Kanonenberg et al. 2018). Since the cytoplasmic Ca^2+^ concentration (~ 100 nM) is below the K_D_ value of this binding event (~ 100 µM), folding happens exclusively after transport in the cell surrounding (Ca^2+^ ≥ 2 mM). In the absence of the T1SS HlyA is not secreted and forms IBs, presumably due to low Ca^2+^ concentration within the cytoplasm forcing aggregation (Bumba et al. 2016). Extracted and denatured IBs of HlyA and the HlyA fragment HlyA1 (aa 806–1024), however, can be renatured in the presence of Ca^2+^ ions (Lecher et al. 2012). Obviously, Ca^2+^ ions are ionic switches transforming aggregating HlyA in highly soluble proteins. We analyzed whether this behavior is conserved in truncated HlyA peptide fusion proteins and whether this approach can be applied as expression platform for peptides and small proteins (Fig. 2).

**Fig. 1:**
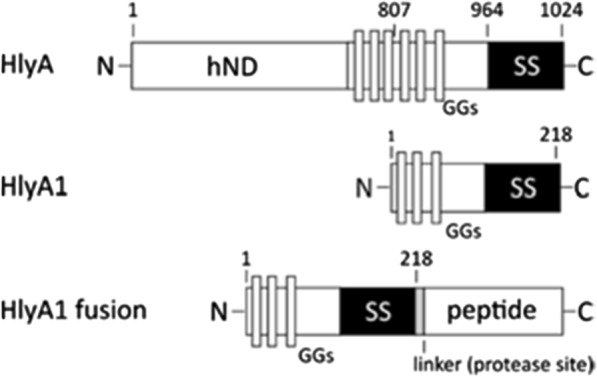
Schematic view of HlyA, HlyA1 and a HlyA1 fusion. HlyA consist of a hydrophobic N-terminal domain (hND), an RTX domain (RTX) characterized by the presence of so-called GG repeats (GGs) and a C terminal secretion signal (SS, ~ 60 aa). In HlyA1 and HlyA1 fusions the C-terminal 218 aa of HlyA are present. Between HlyA1 and fused targets a *Tobacco Edge Virus* (TEV) cleavage site is inserted. Numbers indicate the position in the corresponding amino acid sequence

**Fig. 2:**
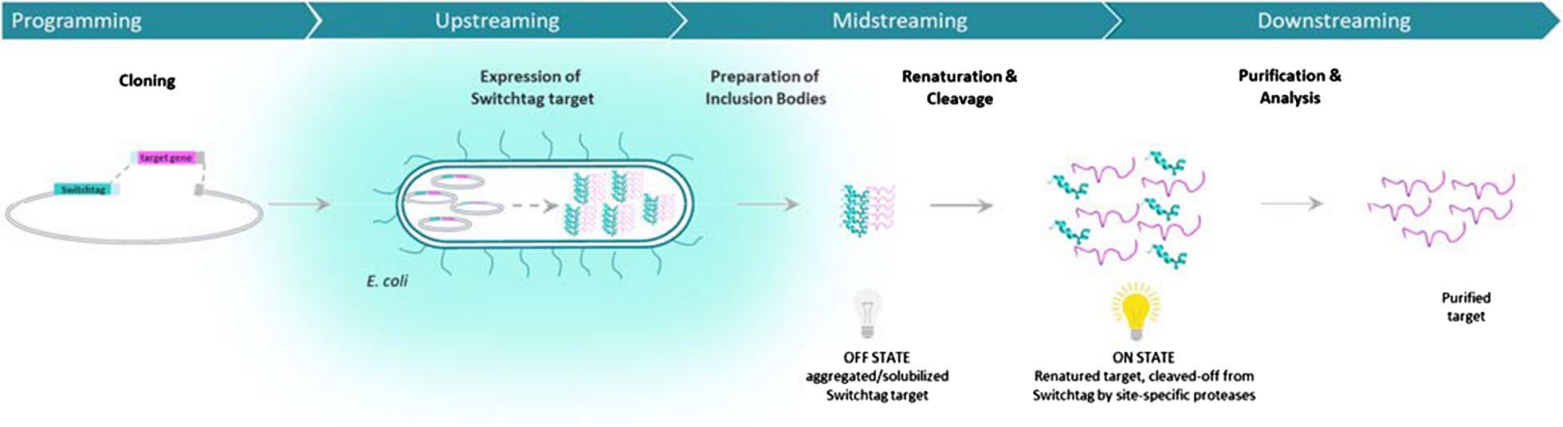
Schematic view of the Numaswitch approach. Being integrated into an expression cassette and expressed in *E. coli*, Switchtag targets form inclusion bodies (IBs) inside the cytoplasm (IB-tag). The IBs are extracted and solubilized. Switchtag targets are subsequently quantitatively renatured in the presence of Ca^2+^ ions (renaturation tag). After renaturation targets are separated from the Switchtag by site-specific proteases releasing traceless products without any non-desired amino acids remaining attached

## Materials and methods

### Cloning, expression and IB preparation

The DNA-sequences encoding for HlyA1 truncations and HlyA1 peptides/small proteins (Table S1) including Teriparatide, Linaclotide, Dermaseptin–Thanatin (DS-THA), human β-amyloid (1–40) (Aβ40) and Serum amyloid A3 (SAA3) were cloned in the parental plasmid of HlyA1 (Khosa et al. 2018). Initial expression studies were performed in *E.* *coli* BL21 (DE3) in shaking flasks at 30 °C for 24 h. IPTG was used for induction of expression and cells were harvested by centrifugation. IB preparation was carried out by Bug Buster Kit (Novagene) according to the manual. Gram scale expression was done by high cell density fermentation using an extended fed-batch approach. For IB preparation cells were disrupted by pressure homogenization (LM-20, Microfluidics). IBs were washed with buffer (Tris/HCl pH 7.3 10 mM, 120 mM NaCl, 2 mM EDTA, 0.1% Triton-X-100) and sedimented by centrifugation. The used protease from the *Tobacco Etch Virus* (TEV) was produced as described elsewhere (Blommel and Fox 2007).

### Renaturation of IBs and TEV protease cleavage

HlyA1, HlyA1 truncations and HlyA1 fusion IBs were solubilized in guanidinium hydrochloride (GuHCl, 6 M) (1:4, w/v). Protein concentrations were determined by UV/Vis spectroscopy using the calculated molecular weights and extinction coefficients (ProtParam, Expasy). The concentration of solubilized proteins were set to 1 mM with 6 M GuHCl. For renaturation studies of HlyA1 and HlyA1 fusions were diluted to 0.05 mM final concentration into renaturation buffer (20 mM Tris/HCl buffer, pH 8, 150 mM NaCl, 0.5 mM EDTA) in absence or presence of 10 mM CaCl_2_. Renaturation reactions were incubated for 20 min at room temperature (RT). Renaturation efficiencies were determined by dividing the protein concentration in the cleared supernatant (centrifugation, 10 min, 13.200×g, RT) by the maximal expected value of the protein concentration adjusted for the renaturation reaction (0.05 mM). Protease cleavage reactions were performed at 30 °C for 3 h after adding TEV protease in a molar ratio of 1:25. Renaturation and cleavage samples were analyzed by SDS-PAGE (15%) stained with Coomassie brilliant blue. RP-HPLC/MS analyses were performed (Alliance QDa detector, Waters, ZORBAX 300SB-C18 column, 4.6 × 250 mm, 5 µm, Agilent) with a water/acetonitrile gradient supplemented with 0.1% trifluoro acetic acid (TFA).

### Production of Teriparatide

HlyA1 ∆165–218 Teriparatide IBs were expressed, extracted, washed as described above. Renaturation was performed in HEPES-based renaturation buffer (pH 8, 20 mM, 10 mM CaCl_2_) adjusting the protein concentration to 2 mg/mL using IBs solubilized in 8 M urea (1:8, w/v). TEV-protease was added in a molar ratio of 1:200 and cleavage reaction was incubated at RT for 3 h. The peptide was purified by cation exchange chromatography (CEX, Capto SP ImpRes resin, Cytiva) using a NaCl gradient in Na-acetate buffer (20 mM, pH 6.5). Teriparatide-containing elution fractions were further purified on a RP FLASH column (Aquarius C18AQ, BGB, water/acetonitrile gradient with 0.1% TFA). TFA/acetic acid exchange was performed by washing the Teriparatide-loaded RP FLASH column in presence of 3% acetic acid. After elution, Teriparatide containing fractions were pooled and lyophilized. Lyophilized sample was analyzed by RP-HPLC/MS, peptide mapping, IC chromatography (counter ions), endotoxin assay (BioChem, Karlsruhe), qPCR for the detection of residual host cell DNA (rHCD) and a commercially available residual host cell protein (rHCP) ELISA (#F410, Cygnus technologies). Functionality was confirmed compared to WHO standard (Charles River Laboratories) (Table 1). Net/gross weight of produced Teriparatide was determined by dissolving a weighted lyophilized fraction in water and determination of the protein concentration in solution by UV/VIS spectroscopy.

**Table 1:** Release analytics of Teriparatide product

Property	Specification	Measured
Identity (4117.7 Da)	1030.4 [M + 4H]^+4^	1030.4 [M + 4H]^+4^
Peptide mapping (aa)	23–30, 23–34, 5–22, 5–19	confirmed
Purity	(≥ 95) %	99.6%
Net/gross weight	> 80	88.7%
Acetate/TFA/Cl	> 95% Acetate	96/3/1 (mol%)
Endotoxins	< 5 EU/mg	< 0.4 EU/mg
rHCP	< 500 ng/mg	< 100 ng/mg
rHCD	< 200 pg/mg	< 10 pg/mg
Functionality	As WHO standard	Confirmed

## Results

### Expression and Renaturation of HlyA1 and HlyA1 fusions

**Fig. 3:**
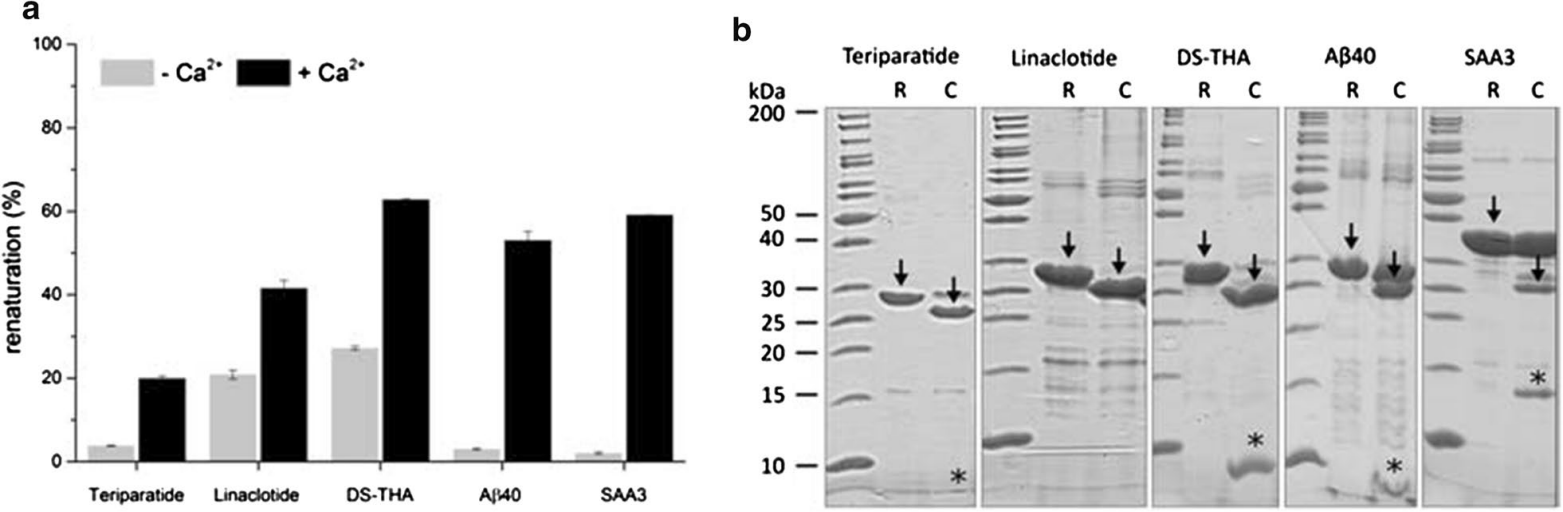
Renaturation efficiencies and TEV protease cleavage of HlyA1 fusions. **a** Renaturation efficiencies (%) of HlyA1 fusions in the presence or absence of 10 mM Ca^2+^ ions. 100% renaturation efficiency corresponds to the adjusted protein concentration for the renaturation reaction. Error bars indicate the SD (n = 3). **b** SDS-PAGE analysis (Coomassie-stained) of renatured HlyA1 fusions prior to and after TEV protease cleavage. Arrows indicate the HlyA1 fusions after renaturation (R) and HlyA1 after TEV protease cleavage (C). Signals were found for each target but Linaclotide. Due to visualization limits of Coomassie staining, the location is highlighted (*). Release of targets was further assessed by RP-HPLC analysis (Additional file 1: Fig. S3)

HlyA1 was expressed as IBs and solubilized IBs were renatured in the absence or presence of 10 mM Ca^2+^ with efficiencies of ~ 25% and ~ 60%, respectively (Additional file 1: Fig. S1). Next, we evaluated whether such behavior is conserved for HlyA1 fusions. Target candidates were chosen that vary in molecular weight (1.5–12.3 kDa), physicochemical characteristics (repetitive, antimicrobial, aggregating, hydrophilic, Cys-containing peptides) and functionalities (antimicrobial peptides, active pharmaceutical ingredients) and fused C-terminally to HlyA1 (Additional file 1: Table S1). All HlyA1 fusions were expressed as IBs (Additional file 1: Fig. S2). Solubilized IBs were renatured in the presence or absence of Ca^2+^ ions. Whereas in the absence of Ca^2+^ ions renaturation efficiencies were between 4 and 25%, they increased in the presence of Ca^2+^ ions to 20–63% (Fig. 3a). Evidently, the presence of Ca^2+^ ions increases renaturation efficiencies of HlyA1 and HlyA1 fusions. Separation of the targets from HlyA1 by TEV protease hydrolysis was assessed and analyzed by SDS-PAGE (Fig. 3b). Each target was cleaved-off, however, with different efficiencies. RP-HPLC/MS analysis revealed the formation of distinct elution signals for each target and the identities of Teriparatide, Linaclotide, DS-THA, and Aβ40 were confirmed by mass spectrometry (Additional file 1: Fig. S3). For SAA3, no mass signals could be detected by the applied method. The data show that HlyA1 can be applied as a bifunctional protein tag (IB-tag and renaturation-tag) and the developed approach (named Numaswitch) serves as reliable production strategy for peptides and small proteins.

### Optimization of renaturation efficiencies

**Fig. 4:**
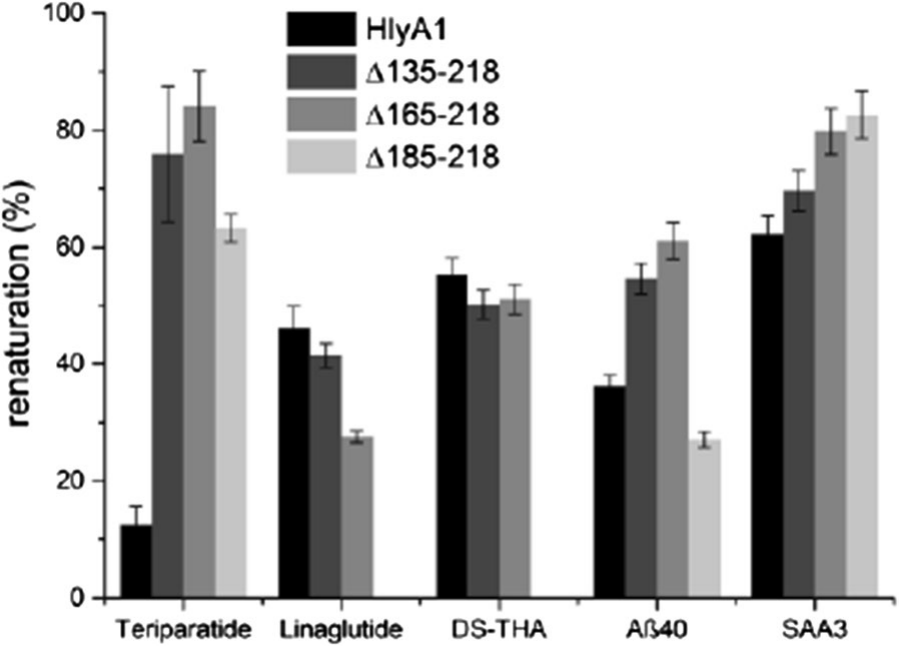
Renaturation efficiencies of HlyA1 fusions and truncated HlyA1 fusion variants. Shown are the renaturation efficiencies (%) in Tris-based buffer in the presence of 10 mM Ca^2+^ ions. Error bars indicate the SD, measurements were performed in triplicates

As next step, we aimed to increase the renaturation efficiencies of HlyA1 fusions. Since it was known from previous results that the C-terminal SS of HlyA1 transfers some degree of instability towards HlyA1, and the RTX domain of HlyA1 was suggested to be the relevant domain for Ca^2+^-assisted refolding (Lecher et al. 2012), three different C-terminal truncations of HlyA1 were generated lacking partially (HlyA1 ∆185–218) or entirely (HlyA1 ∆165–218) the SS or containing a longer C-terminal depletion (HlyA1 ∆135–218). The three truncated HlyA1 variants were fused with the above-mentioned targets. All truncated HlyA1 fusions were expressed (Additional file 1: Fig. S4) and IBs were extracted and renatured. Remarkably, renaturation efficiencies further increased 6.8-fold for Teriparatide (84%), 1.7-fold for Aβ40 (61%) and 1.3-fold for SAA3 (80%) (Fig. 4). For DS-THA the renaturation efficiency did not change significantly and for Linaclotide renaturation efficiencies decreased with all truncated HlyA1 variants. Since HlyA1 ∆165–218 fusions were expressed in high levels and renaturation efficiencies were equal or superior in four out of five constructs, we focused on this variant and named it Switchtag.

### Gram scale production of Teriparatide

**Fig. 5:**
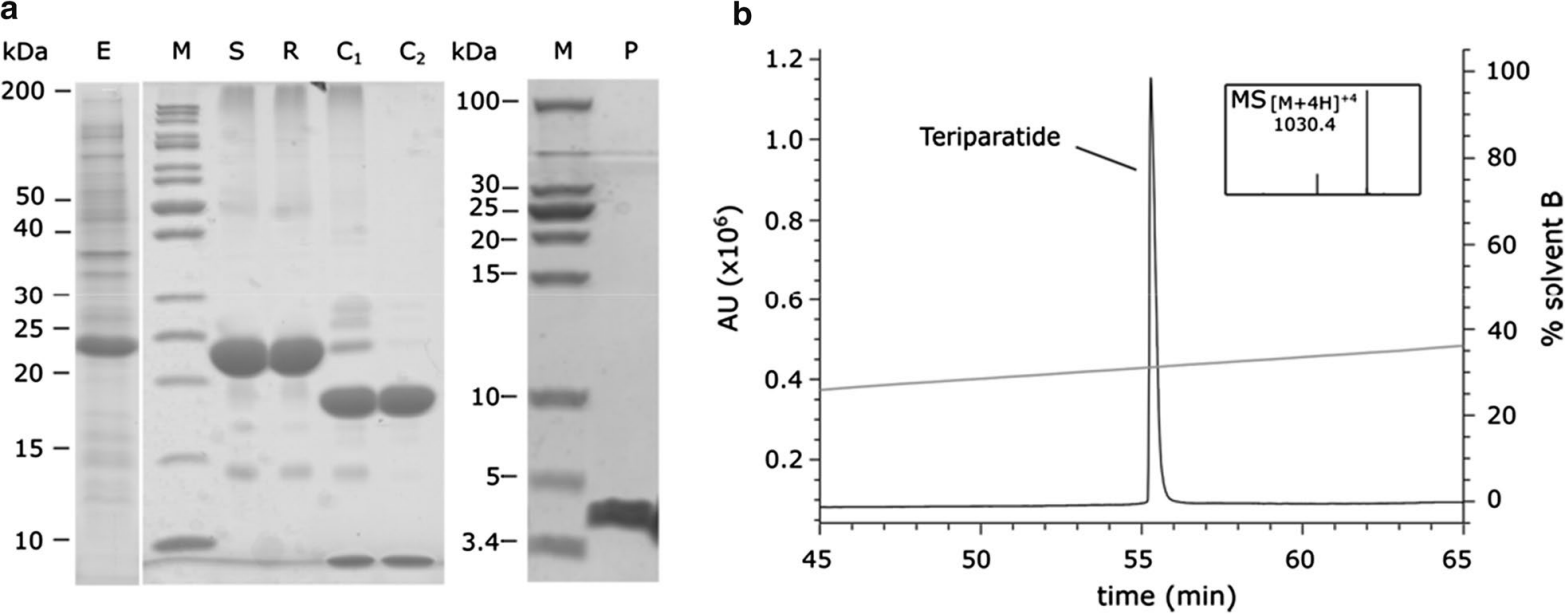
Numaswitch applied to produce Teriparatide **a** SDS-PAGE analysis of different process steps; *E. coli* cells from high cell density fermentation (E), solubilized Switchtag Teriparatide IBs (S), renaturation reaction (R), TEV protease cleavage (C1: crude, C2, cleared) and produced Teriparatide after CEX, RP FLASH purification and TFA/acetate exchange (P). M: Molecular weight marker with shown masses. **b** HPLC/MS analysis of purified Teriparatide revealed > 99.6% purity of lyophilized Teriparatide

The applicability of the Numaswitch approach at pilot scale was evaluated for Teriparatide, a block buster peptide for the treatment of osteoporosis (Minisola et al. 2019). A high cell density fermentation protocol was established (data not shown) yielding 21 g Switchtag Teriparatide IBs net weight per liter fermentation broth (63 g wet weight). Under optimized conditions with HEPES-based renaturation buffer Switchtag Teriparatide was renatured quantitatively (> 95%). Also, the separation of Teriparatide from the Switchtag by TEV protease was quantitative (> 95%) within 3 h (Fig. 5a). Teriparatide was purified by CEX reaching a purity level of > 99% (data not shown).

RP FLASH chromatography was applied for further purification, salt removal and TFA/acetate exchange before lyophilization. The purity of produced Teriparatide was determined by HPLC to be > 99.6% (Fig. 5b). Additional release analytics were performed for functionality, net/gross weight, identity (MS and peptide mapping, (Additional file 1: Fig. S6), counter ion content, endotoxins, rHCD and rHCP (Table 1). From 1 l fermentation broth > 2 g Teriparatide was produced meeting specifications of active pharmaceutical ingredients for human applications.

## Discussion

Numerous expression strategies for the production of proteins are available and product titers of 20 g per liter fermentation broth are described (Tripathi and Shrivastava 2019). Being applied successfully also for peptides in various cases, the development of recombinant production processes is cumbersome adding a time and price tag, particularly due to intrinsic characteristics of peptides including proteolytic sensitivity, degradation, aggregation and cytotoxicity. No reliable expression platform for peptides exists making chemical synthesis the method of choice in most cases.

In this study, HlyA1 fusions and truncated HlyA1 fusions were generated, analyzed and optimized as bifunctional protein tags to produce peptides and small proteins. The first functionality (IB tag) allows the high yield expression of HlyA1 fusions without proteolytical damage or other degradations independent of lengths, physicochemical properties and functionalities granting access to high peptide levels with good initial purities > 85% (Fig. 5a, lane S) and protecting the producer cell from cytotoxic target functionalities. The second functionality (Ca^2+^-assisted renaturation) allows up to quantitative renaturation of IBs and grants access to water-soluble, renatured (truncated) HlyA1 fusions at high purities (> 95%, Fig. 5a, lane C2) decreasing downstreaming efforts and process complexity. Targets can be cleaved-off from HlyA1 and HlyA1 fragments by site-specific proteases, i.e., TEV protease to produce traceless targets without any non-desired amino acid left.

Our studies showed that HlyA1 ∆165–218 (Switchtag) was the best of HlyA1 truncations tested and showed best renaturation efficiencies in 4 of 5 cases. Overall, deletion of the C-terminal SS seems to increase renaturation efficiencies. This is in line with previous observations where HlyA1 fragments lacking a C-terminal portion have increased solubility characteristics (Lecher et al. 2012). Remarkably, renaturation concentrations of Switchtag targets can be as high as several g/L keeping volumes low needed for renaturation reactions. With the Numaswitch approach > 2 g Teriparatide per liter fermentation broth in API quality was produced. Compared to state-of-the-art production strategies (Abbaszadeh et al. 2018) this represents a ~ 20-fold yield increase. Simultaneously, usage of affinity chromatography and HPLCs is avoided making this process scalable and cost-efficient. With Numaswitch, the advantages of IBs become accessible (titer, purity, protection) and, simultaneously the major problem with using IBs, namely the inefficient renaturation, is solved. Since Numaswitch relies on the formation of IBs it can be applied independently of a specific *E. coli* strain and expression host, as long as IBs are formed. Further developments are currently ongoing to analyze the applicability of Numaswitch towards proteins > 120 aa. Also, alternative proteins of the huge RTX family containing several thousands of members are analyzed accordingly.

The Numaswitch approach serves as a reliable high yield expression platform for peptides and small proteins. It thus allows for the production of known, but also the development of new peptides for pharmaceutical applications and beyond by enabling their reliable and cost-efficient production at larger scales and with high qualities.

## Supplementary Information

**Additional file 1: Fig. S1. **Expression of HlyA1 in *E. coli *and renaturation in presence and absence of Ca^2+^ ions. A SDS-PAGE analysis of *E. coli *cells before (−) and after (+) induction of the expression and of the renaturation reaction. Arrows indicate HlyA1 (24 kDa). B Quantification of time-dependent renaturation efficiencies in the absence and presence of Ca^2+^ ions. Error bars indicate the SD, measurements were performed in triplicates. **Table S1. **Name, primary structure and molecular weight (MW) of chosen peptides/small proteins candidates. **Fig. S2.** Expression analysis of HlyA1 fusions*. *SDS-PAGE analysis of *E. coli *cells before (−) and after (+) expression induction of HlyA1 fusions. Arrows indicate the expressed proteins (HlyA1 Teriparatide, 28.0 kDa, HlyA1 Linaclotide, 26.4 kDa, HlyA1 DS-THA, 30.1 kDa, HlyA1 aβ40, 29.2 kDa, HlyA1 SAA3, 37.2 kDa). HlyA1 fusions were present in the insoluble fractions of Bug Buster Kit preparation indicating the expression as IBs. **Fig. S3. **RP HPLC/MS analysis of HlyA1 fusion TEV cleavage reactions. Analysis was performed as described in the material and methods section. The retention time of HlyA1 varied as different water/acetonitrile gradients were used to achieve optimal peak separation. Molecular masses of the elution signals were determined after electron spray ionization and by quadrupole measurement (QDa, Waters). Chromatograms show the UV absorption at 205 nm. **Fig. S4. **Expression analysis of truncated HlyA1 fusions*. *SDS-PAGE analysis of *E. coli *cells before (−) and after (+) expression induction. Arrows indicate the expressed truncated HlyA1 fusions in the Coomassie-stained gel. For Linaclotide and DS-THA cloning of the truncated variant Δ185–218 failed. **Fig. S5. **Renaturation and protease cleavage of truncated HlyA1 fusions. Analysis of the renaturation and protease cleavage reaction by SDS-PAGE (Coomassie-stained). Arrows indicate the HlyA1-backbones (Δ135–218, Δ165–218 or Δ185–218) after protease cleavage (C). The released target is indicated (*). Linaclotide release was confirmed by HPLC/MS although it was not visible on the Coomassie-stained gel. **Fig. S6**. Peptide mapping of produced Teriparatide in comparison to commercially available drugs containing Teriparatide. The produced Teriparatide was compared in peptide mapping experiments to the commercial drug products FORSTEO® (Lilly) and TERROSA® (Gedeon Richter). Endopeptidase Glu-C (Sigma-Aldrich) was used for Teriparatide digestion and the digestion products were analyzed by RP-HPLC/MS analysis. The indicated peaks (215 nm) correspond to the Glu-C digestion products of Teriparatide aa 23–30, aa 23–34, aa 5–19 and aa 5–22.

## Data Availability

All data generated and analyzed during this study are included in this published article and its Additional file. If additional information is required please contact the corresponding author.
