# Supplementary material for: Numaswitch: an efficient high-titer expression platform to produce peptides and small proteins
Source: AMB Express. 2021 Mar 25;11:48. doi: 10.1186/s13568-021-01204-w (PMC7994495; doi:10.1186/s13568-021-01204-w)
Supplement: Supplementary file 1 — Additional file 1: Fig. S1. Expression of HlyA1 in E. coli and renaturation in presence and absence of Ca2+ ions. A SDS-PAGE analysis of E. coli cells before (−) and after (+) induction of the expression and of the renaturation reaction. Arrows indicate HlyA1 (24 kDa). B Quantification of time-dependent renaturation efficiencies in the absence and presence of Ca2+ ions. Error bars indicate the SD, measurements were performed in triplicates. Table S1. Name, primary structure and molecular weight (MW) of chosen peptides/small proteins candidates. Fig. S2. Expression analysis of HlyA1 fusions. SDS-PAGE analysis of E. coli cells before (−) and after (+) expression induction of HlyA1 fusions. Arrows indicate the expressed proteins (HlyA1 Teriparatide, 28.0 kDa, HlyA1 Linaclotide, 26.4 kDa, HlyA1 DS-THA, 30.1 kDa, HlyA1 aβ40, 29.2 kDa, HlyA1 SAA3, 37.2 kDa). HlyA1 fusions were present in the insoluble fractions of Bug Buster Kit preparation indicating the expression as IBs. Fig. S3. RP HPLC/MS analysis of HlyA1 fusion TEV cleavage reactions. Analysis was performed as described in the material and methods section. The retention time of HlyA1 varied as different water/acetonitrile gradients were used to achieve optimal peak separation. Molecular masses of the elution signals were determined after electron spray ionization and by quadrupole measurement (QDa, Waters). Chromatograms show the UV absorption at 205 nm. Fig. S4. Expression analysis of truncated HlyA1 fusions. SDS-PAGE analysis of E. coli cells before (−) and after (+) expression induction. Arrows indicate the expressed truncated HlyA1 fusions in the Coomassie-stained gel. For Linaclotide and DS-THA cloning of the truncated variant Δ185–218 failed. Fig. S5. Renaturation and protease cleavage of truncated HlyA1 fusions. Analysis of the renaturation and protease cleavage reaction by SDS-PAGE (Coomassie-stained). Arrows indicate the HlyA1-backbones (Δ135–218, Δ165–218 or Δ185–218) after protease cleavage (C). The rele [file 13568_2021_1204_MOESM1_ESM.pdf]

# Numaswitch – an efficient high-titer expression platform to produce peptides and small proteins

Bach-Ngan Nguyen[1,2], Florian Tieves[1], Thomas Rohr[1,5], Hilke Wobst[1], Felix S. Schöpf[1], Jóse D. Montoya Solano[1], Julia Schneider[1], Janpeter Stock[1], Andreas Uhde[1], Thomas Kalthoff[1], Karl Erich Jaeger[3,4], Lutz Schmitt[2], Christian Schwarz[1]*

## HlyA1 renaturation in the presence and absence of Ca2+ ions

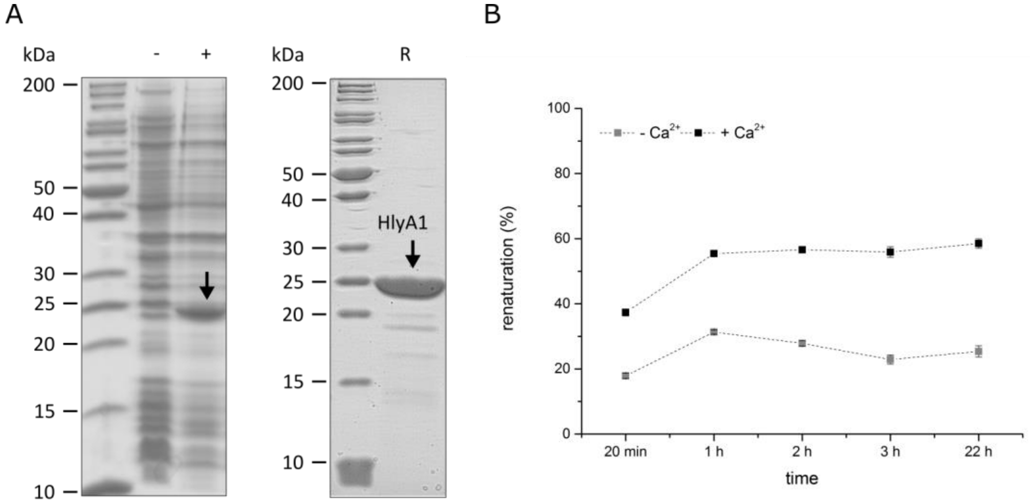


**Figure S1 Expression of HlyA1 in *E. coli* and renaturation in presence and absence of $Ca^{2+}$ ions.** A SDS-PAGE analysis of *E. coli* cells before (-) and after (+) induction of the expression and of the renaturation reaction. Arrows indicate HlyA1 (24 kDa). B Quantification of time-dependent renaturation efficiencies in the absence and presence of $Ca^{2+}$ ions. Error bars indicate the SD, measurements were performed in triplicates.

**Teriparatide, Linaclotide, DS-THA, Aβ40 and SAA3 were chosen as peptide/small protein candidates varying in lengths, physicochemical characteristics and functionalities (Table S1)**

**Table S1. Name, primary structure and molecular weight (MW) of chosen peptides/small proteins candidates.**

| Peptide name | Primary structure (one letter code) | MW (kDa) |
|---|---|---|
| Teriparatide | SVSEIQLMHNLGKHLNSMERVEWLRKKLQDVH NF | 4.2 |
| Linaclotide | CCEYCCNPACTGCY | 1.5 |
| Dermaseptin-Thanatin (DS-THA) | GLWSTIKQKGKEAAIAAAKAAGQAALGALGSK KPVPIIYCNRRTGKCQRM | 5.2 |
| Human β-amyloid (1-40) (Aβ40) | DAEFRHDSGYEVHHQKLVFFAEDVGSNKGAIIG LMVGGVV | 4.3 |
| Serum amyloid A3 (SAA3) | QGWGTFLREAGQGAKDMWRAYRDMKEANYK GADKYFHARGNYDAAQRGPGGVWAAEVIREA LQGITDPLFKGMTRPQVREDTKADQFANEWGQ SGKDPNHFGPAALPDKY | 12.3 |

## Small scale expression of HlyA1 fusions

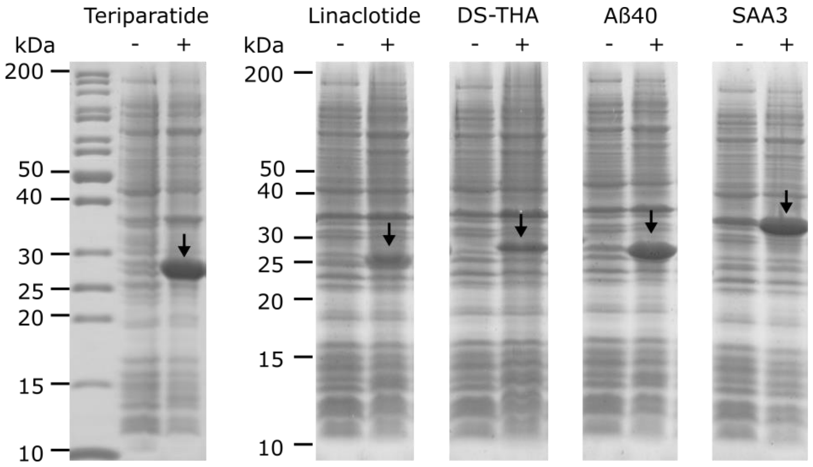


**Figure S2 Expression analysis of HlyA1 fusions. SDS-PAGE analysis of *E. coli* cells before (-) and after (+) expression induction of HlyA1 fusions.** Arrows indicate the expressed proteins (HlyA1 Teriparatide, 28.0 kDa, HlyA1 Linaclotide, 26.4 kDa, HlyA1 DS-THA, 30.1 kDa, HlyA1 aβ40, 29.2 kDa, HlyA1 SAA3, 37.2 kDa). HlyA1 fusions were present in the insoluble fractions of Bug Buster Kit preparation indicating the expression as IBs.

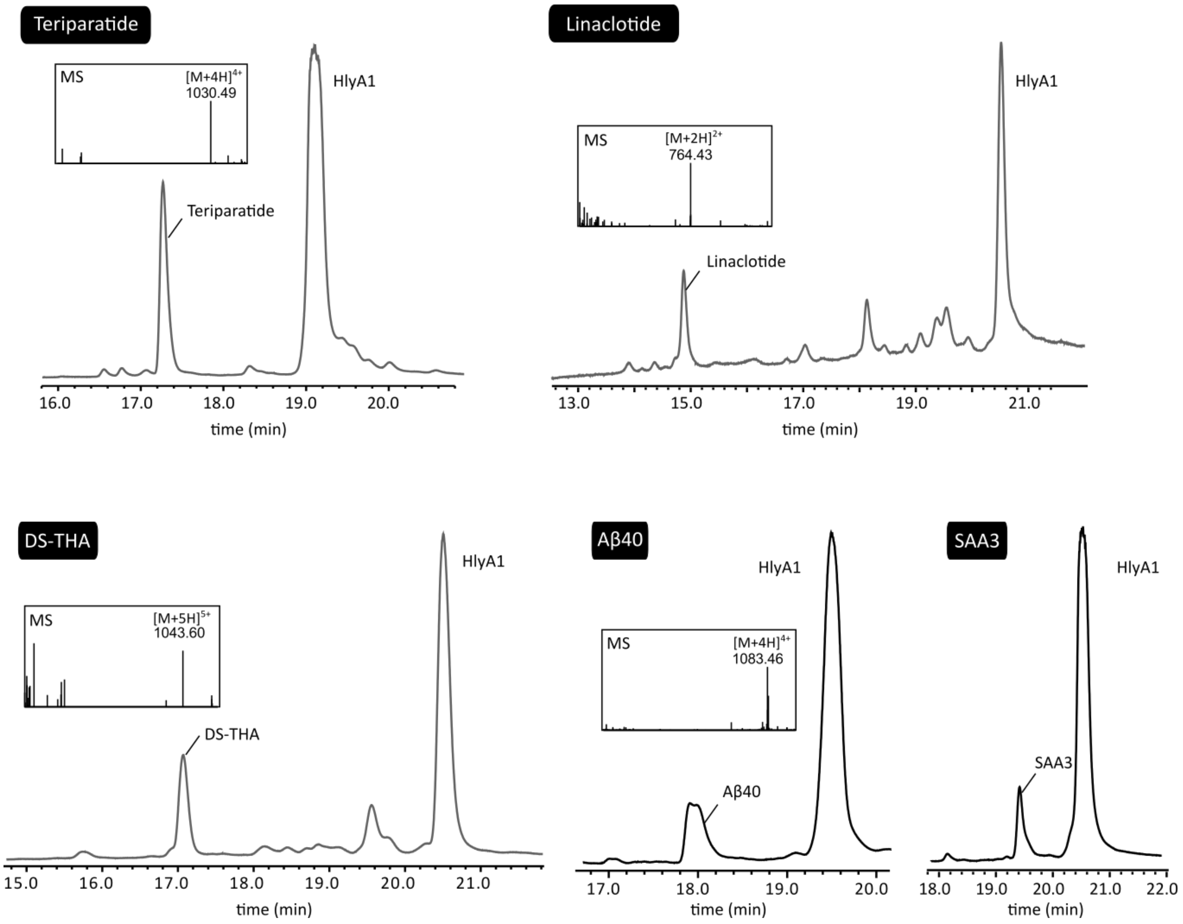


**Figure S3 RP HPLC/MS analysis of HlyA1 fusion TEV cleavage reactions. Analysis was performed as described in the material and methods section.** The retention time of HlyA1 varied as different water/acetonitrile gradients were used to achieve optimal peak separation. Molecular masses of the elution signals were determined after electron spray ionization and by quadrupole measurement (QDa, Waters). Chromatograms show the UV absorption at 205 nm.

## Expression of truncated HlyA1 fusions

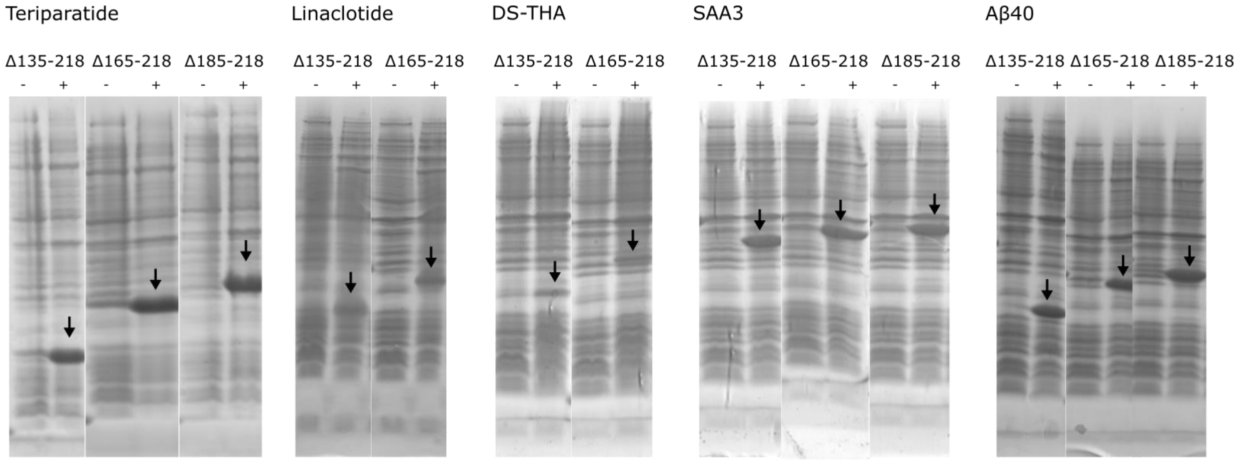


**Figure S4 Expression analysis of truncated HlyA1 fusions.** SDS-PAGE analysis of *E. coli* cells before (-) and after (+) expression induction. Arrows indicate the expressed truncated HlyA1 fusions in the Coomassie-stained gel. For Linaclotide and DS-THA cloning of the truncated variant Δ185-218 failed.

## Renaturation and TEV protease cleavage of truncated HlyA1 fusions.

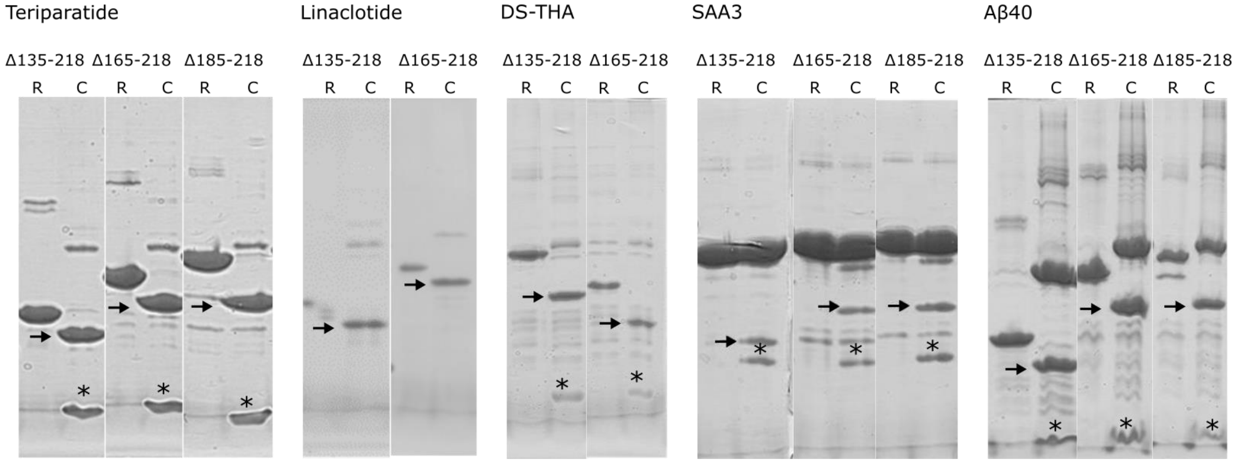


**Figure S5. Renaturation and protease cleavage of truncated HlyA1 fusions.** Analysis of the renaturation and protease cleavage reaction by SDS-PAGE (Coomassie-stained). Arrows indicate the HlyA1-backbones (Δ135-218, Δ165-218 or Δ185-218) after protease cleavage (C). The released target is indicated (*). Linaclotide release was confirmed by HPLC/MS although it was not visible on the Coomassie-stained gel.

## Peptide mapping of Teriparatide product and commercial drugs

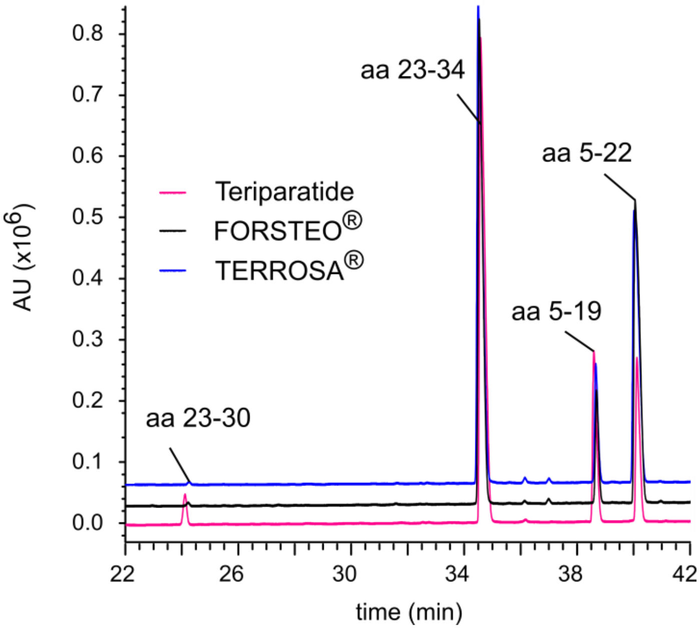


**Figure S6. Peptide mapping of produced Teriparatide in comparison to commercially available drugs containing Teriparatide.** The produced Teriparatide was compared in peptide mapping experiments to the commercial drug products FORSTEO® (Lilly) and TERROSA® (Gedeon Richter). Endopeptidase Glu-C (Sigma-Aldrich) was used for Teriparatide digestion and the digestion products were analyzed by RP-HPLC/MS analysis. The indicated peaks (215 nm) correspond to the Glu-C digestion products of Teriparatide aa 23-30, aa 23-34, aa 5-19 and aa 5-22.
